# Modification of the SnO_2_ Electron Transporting Layer by Using Perylene Diimide Derivative for Efficient Organic Solar Cells

**DOI:** 10.3389/fchem.2021.703561

**Published:** 2021-06-25

**Authors:** Tianyu Kong, Rui Wang, Ding Zheng, Junsheng Yu

**Affiliations:** State Key Laboratory of Electronic Thin Films and Integrated Devices, School of Optoelectronic Science and Engineering, University of Electronic Science and Technology of China (UESTC), Chengdu, China

**Keywords:** organic solar cell, electron transporting layer, perylene diimide derivative, tin oxide, surface defects

## Abstract

Recently, tin oxide (SnO_2_) nanoparticles (NPs) have attracted considerable attention as the electron transporting layer (ETL) for organic solar cells (OSCs) due to their superior electrical properties, excellent chemical stability, and compatibility with low-temperature solution fabrication. However, the rough surface of SnO_2_ NPs may generate numerous defects, which limits the performance of the OSCs. In this study, we introduce a perylene diimide derivative (PDINO) that could passivate the defects between SnO_2_ NP ETL and the active layer. Compared with the power conversion efficiency (PCE) of the pristine SnO_2_ ETL–based OSCs (12.7%), the PDINO-modified device delivers a significantly increased PCE of 14.9%. Overall, this novel composite ETL exhibits lowered work function, improved electron mobility, and reduced surface defects, thus increasing charge collection efficiency and restraining defect-caused molecular recombination in the OSC. Overall, this work demonstrates a strategy of utilizing the organic–inorganic hybrid ETL that has the potential to overcome the drawbacks of SnO_2_ NPs, thereby developing efficient and stable OSCs.

## Introduction

Over the past decades, in order to harness clean and abundant solar energy, extensive efforts have been made to develop efficient and affordable photovoltaic cells. Among the numerous candidates, organic solar cell (OSC) has attracted considerable attention of researchers due to its potential of low-cost and large-scale fabrication onto the flexible or stretchable substrate (Thompson and Frechet, 2008; Liang et al., 2010; Li et al., 2012; Hou et al., 2018; Yuan et al., 2019). Owing to the continuous development of organic photoelectric materials in recent years, for the bulk heterojunction (BHJ) OSC device, the power conversion efficiency (PCE) has exceeded 18% (Wang et al., 2020), which paves the way for the future commercialization of OSCs. In addition to the active layer, the charge transporting layer (CTL) also plays a critical role in realizing the high-performance of OSCs (Ma et al., 2010; Yip and Jen, 2012). Being inserted between the electrode and active layer, the CTL can decrease the interfacial barrier and adjust the mismatched energy levels, thus facilitating charge carrier collection and transportation (Hsieh et al., 2010; Li et al., 2010). Currently, an n-type metal oxide, namely, zinc oxide (ZnO), has been widely utilized as the material of the electron transporting layer (ETL) for OSCs because of its matched energy level, good conductivity, high optical transparency, and solution processability (White et al., 2006; Kyaw et al., 2008; Wang et al., 2015; Zhang et al., 2019; Zheng et al., 2019; Fan et al., 2020). However, when placed under ambient sun illumination, ZnO can absorb a large proportion of ultraviolet light, which brings about the degradation of the organic active layer and thus hampers the performance of OSCs (Jiang et al., 2019).

Tin oxide (SnO_2_) is another n-type metal oxide that can be fabricated from pre-dispersed nanoparticle (NP) dispersion annealed at a temperature of less than 150 °C. It has been widely studied as the ETL material in dye-sensitized and perovskite solar cells (Snaith and Ducati, 2010; Jiang et al., 2017). SnO_2_ NPs have a wider bandgap, higher conductivity, and less sensitivity to ultraviolet light than the conventional sol-gel ZnO. However, despite the potential to become a superior ETL material for more stable and efficient OSCs, the solution-based fabrication process of the SnO_2_ NP ETL inevitably generates a mass of surface defects (Peng et al., 2020). Additionally, the morphology of the active layer may be further affected by the compatibility issue with organic photovoltaic materials and metal oxide NPs (Yin et al., 2016). Given the above, the introduction of the SnO_2_ NP ETL could lead to the declines of exciton dissociation and charge extraction efficiencies as well as defect-caused molecular recombination, which significantly limits the performance of OSCs.

Several efforts of surface modification for metal oxide ETLs have been made using various materials, including aluminum (Lin et al., 2016), graphene (Gollu et al., 2016), quantum dot (Zeng et al., 2017), conjugated polyelectrolyte (Kim et al., 2015), and organic small molecule (Song et al., 2013). Among them, perylene diimide (PDI) derivatives, a group of organic small molecule material, have recently drawn lots of research interest due to their excellent stability and the ability to facilitate charge carrier transport by tuning the work function between the active layer and electrodes (Ling et al., 2007; Zhang et al., 2014; Wang et al., 2017). Moreover, the perylene diimide backbone can be easily functionalized by different side chains, thus enabling its water/alcohol processability. A PDI derivative with amine functional groups (PDIN) was utilized as a ZnO ETL modifier, resulting in a near 14% enhanced PCE (Yu et al., 2016). More recently, (HOOC_5_-triazole) PDIN-hex, a carboxylic acid functionalized PDI, was also successfully adopted. The deposition of functionalized PDI can increase the surface hydrophobicity of the ZnO ETL without causing severe impacts to its optical and photochemical properties, thus accumulating the acceptor component of BHJ at the cathode interface. On account of the enhanced charge carrier concentration, the OSC devices with the modified ZnO ETL exhibited up to 33% improvement in the PCE (Abd-Ellah et al., 2019). Despite these benefits of the PDI derivatives on ZnO, their effects on SnO_2_ ETL–based OSCs have not been reported so far.

In this work, a thin layer of perylene diimide derivative with the terminal substituents of amino N-oxide {i.e., 2,9-bis [3-(dimethyloxidoamino) propyl] anthra (2,1,9-def:6,5,10-days'e'f') diisoquinoline-1,3,8,10(2H,9H)-tetrone, (PDINO)} was introduced to modify the SnO_2_ NP ETL. This SnO_2_/PDINO composite ETL exhibits decent optical transmittance, improved electrical conductivity, and reduced work function (WF). Furthermore, the decline of the surface defects is featured prominently in solution-processed SnO_2_ NPs, resulting in an active layer film with more favorable morphology atop the ETL. The poly {[2.6-(4,8-bis(5-(2-ethylhexyl-3-fluoro) thiophen-2-yl)-benzo (1,2-b:4,5-b’) dithiophene)]-alt-[5.5-(1′,3′-di-2-thienyl-5′,7′-bis(2-ethylhexyl)benzo (1′,2′-c:4′,5′-c’) dithiophene-4,8-dione)]}:2.2'-{(2Z,2′Z)-[(12,13-bis(2-ethylhexyl)-3,9-diundecyl-12,13-dihydro-(1,2,5) thiadiazolo (3,4-e) thieno (2″,3’':4′,5') thieno (2′,3':4.5) pyrrolo (3,2-g) thieno (2′,3':4.5) thieno (3,2-b) indole-2,10-diyl) bis(methanylylidene)] bis (5,6-difluoro-3-oxo-2,3-dihydro-1H-indene-2,1-diylidene)}dimalononitrile (PM6:Y6) OSCs were fabricated with a inverted device structure, and the champion device with SnO_2_/PDINO ETLs showed excellent power conversion efficiency (PCE) of 14.9%, which is much higher than that of the control device with the neat SnO_2_ ETL (12.7%). The simultaneously enhanced short circuit current (*J*_*SC*_) and fill factor (FF) indicate that modifying the surface of the SnO_2_ ETL with PDINO could increase charge collection efficiency and restrain defect-caused molecular recombination in the OSC. The device stability also benefits from the PDINO modification. This novel method provides a promising approach to produce efficient OSCs based on low-temperature fabrication.

## Materials and Methods

**FIGURE 1:**
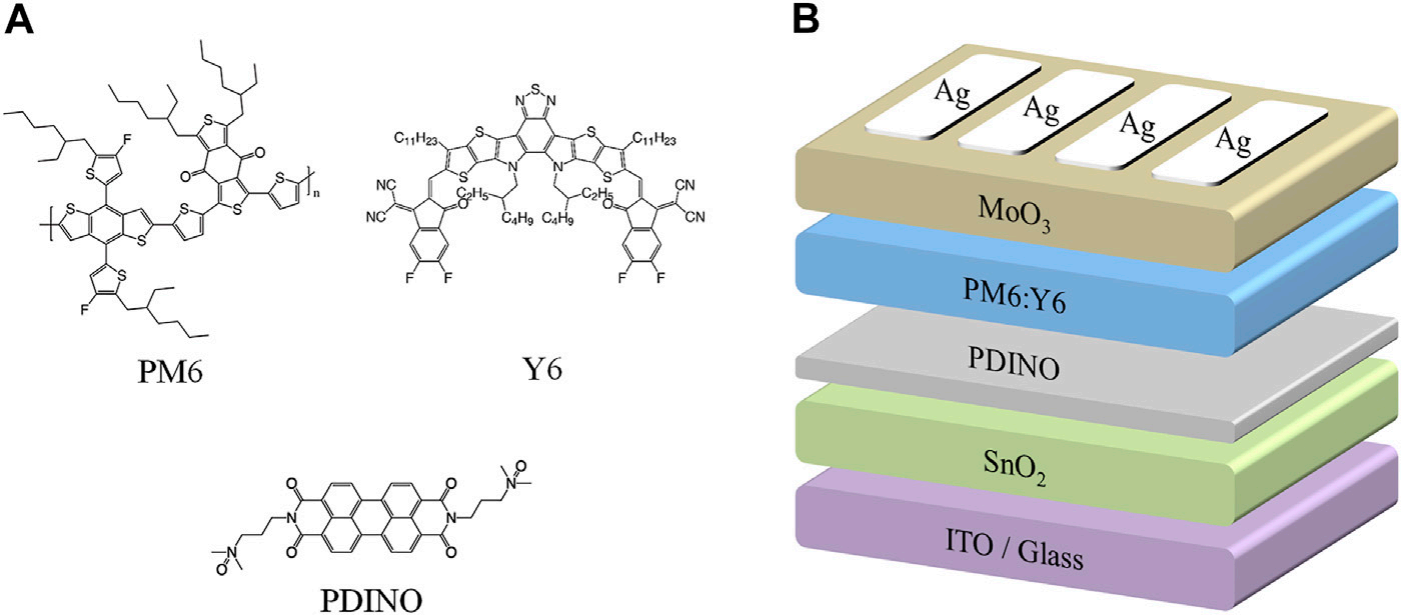
**(A)** Molecular structures of organic photoelectric materials used in this study. **(B)** Schematic diagram of the OSC device structure.

The active layer materials (PM6 and Y6) were purchased from Solarmer, PDINO was obtained from 1-Material, and SnO_2_ NPs were obtained from Alfa Aesar. All of these materials were used as received. The molecular structures of PM6, Y6, and PDINO are shown in Figure 1A. In addition, chloroform, 1-chloronaphthalene, and other solvents used for device fabrication were purchased from Sigma-Aldrich.

The device structure of the OSCs in this study is ITO/ETLs/PM6:Y6/MoO_3_/Ag, as depicted in Figure 1B. With the weight ratio of 1:1.2, PM6 and Y6 were stirred in chloroform for 12 h to obtain the BHJ active layer solution. The blend solution has a concentration of 16 mg/ml; 1-chloronaphthalene at a concentration of 0.5% (v/v) was subsequently mixed as solvent additive. The active layer solution was stirred overnight at room temperature. The SnO_2_ NP ETL (40 nm) was prepared by spin-coating the pre-dispensed SnO_2_ NPs onto cleaned ITO/glass substrates at 5,000 rpm for 45 s and then baked in the air at 120°C for 15 min. For the modified devices, methanol solutions of PDINO at varying concentrations were deposited atop SnO_2_ ETLs at 3,000 rpm for 30 s; the concentration of PDINO is 0.5, 1, and 2 mg/ml. The active layers (100 nm) were spin-coated at 2,000 rpm for 60 s atop the ETLs, followed by a 10-min thermal annealing process in nitrogen glove box at 110°C. Finally, the MoO_3_ hole transporting layer (15 nm) and Ag electrodes (100 nm) were deposited under a vacuum of 1 × 10^−5^ Pa. The effective device area is 0.03 cm^2^, which is determined by the shadow mask.

## Results and Discussion

**FIGURE 2:**
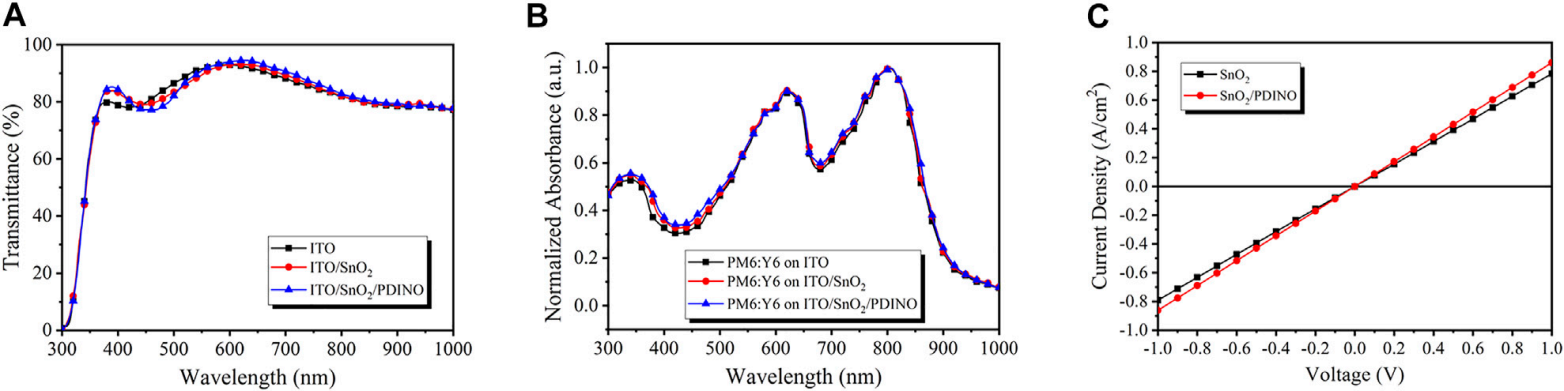
**(A)** Transmittance spectra of ITO, ITO/SnO_2_, and ITO/SnO_2_/PDINO. **(B)** Normalized UV–vis absorption spectra of PM6:Y6 on ITO, ITO/SnO_2_, and ITO/SnO_2_/PDINO. **(C)** *J*–*V* curves of ETLs with and without PDINO modification.

The performance of the inverted OSC device is crucially influenced by the optical property of the ETL. Therefore, the transmittance spectra of ITO-coated glass, SnO_2_, and SnO_2_/PDINO ETLs fabricated on ITO/glass were measured and plotted in Figure 2A. Compared to the clean ITO/glass, SnO_2_ and SnO_2_/PDINO ETLs both exhibit enhanced transmittance in the 350–450 nm region that could be beneficial for achieving better absorption of the active layer. SnO_2_ covered by the PDINO modification layer has slightly lower transmittance from 450 to 550 nm, which could be attributed to the light absorption of PDINO. Despite this result, the SnO_2_/PDINO ETL can still maintain an average transmittance of 87.33% in the 380–780 nm region of visible light, indicating that the PDINO layer will not remarkably impact the light-harvesting ability of the SnO_2_ ETL. The normalized ultraviolet–visible (UV-vis) absorption spectra of PM6:Y6 films on different ETLs are displayed in Figure 2B. The PM6:Y6 films on ITO/SnO_2_ and ITO/SnO_2_/PDINO exhibit higher absorbance than those deposited on the ITO substrate in the wavelength of 350–450 nm, which are consistent with the results of the transmittance spectra in Figure 2A.

The film conductivities of different ETLs were evaluated to take a glimpse into the effect of the PDINO modification layer. With the ITO/ETL/Ag structure to ensure reliable ohmic contact, the current density–voltage (*J*–*V*) measurements of SnO_2_ and SnO_2_/PDINO ETLs are depicted in Figure 2C. The conductivity (*σ*) of the ETLs is defined with Eq. 1:

$$\sigma =\frac{Gd}{A}=\frac{Id}{VA}=\frac{Jd}{V}.$$ (1)

The conductance (G) is calculated from the slope of the *J*–*V* lines, where *A* is the device area, *J* is the current density, *V* is the corresponding bias voltage, and *d* is the thickness of the ETL (*d* = 40 nm for SnO_2_ and *d* = 50 nm for SnO_2_/PDINO). The conductivity of SnO_2_ and SnO_2_/PDINO ETLs is calculated to be 3.10 × 10^−6^ and 4.31 × 10^−6^ S/cm, respectively. The enhanced conductivity of the SnO_2_/PDINO ETL suggests that the modified ETL may have fewer surface defects, thus facilitating electronic transmission.

**FIGURE 3:**
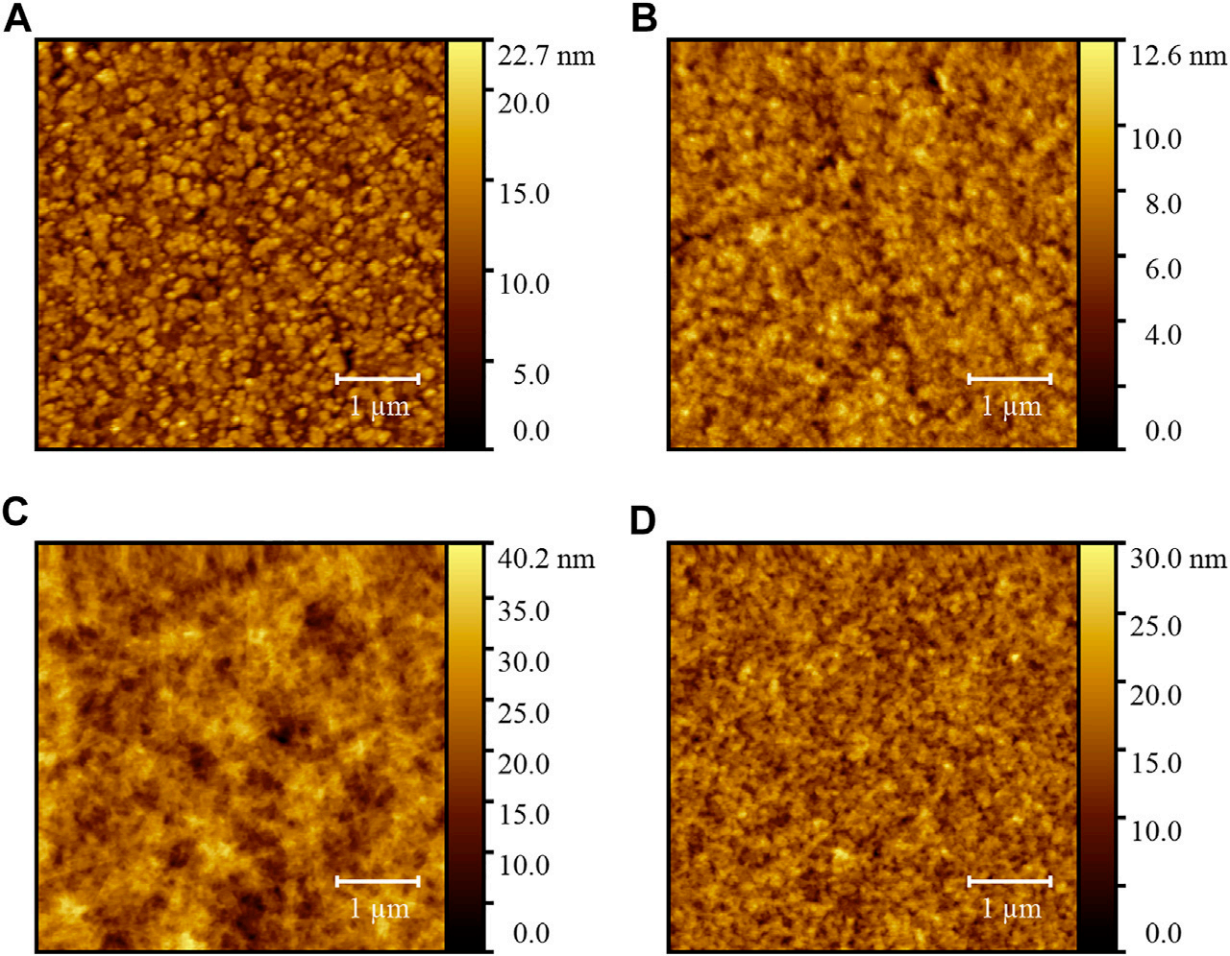
**(A)** AFM height image of the SnO_2_ ETL film. **(B)** AFM height image of the SnO_2_/PDINO ETL film. **(C)** AFM height image of the PM6:Y6 blend film on the SnO_2_ ETL. **(D)** AFM height image of the PM6:Y6 blend film on the SnO_2_/PDINO ETL.

Atomic force microscopy (AFM) was conducted to survey the evolution of surface morphology for the PDINO-modified SnO_2_ NP ETL. Figures 3A,B display the height images of the SnO_2_ ETL and SnO_2_/PDINO ETL. The pristine SnO_2_ ETL film has a relatively coarse surface, with a 2.71-nm root mean square (RMS) roughness value. For the SnO_2_ ETL modified by a PDINO layer, the AFM image exhibits a smoother surface of the ETL, with a significantly decreased RMS roughness value of 1.41 nm. This implies that PDINO could fill the gaps between the SnO_2_ NP aggregates. In order to further elucidate the effects of PDINO modification, the height images of the PM6:Y6 BHJ blend films fabricated atop different ETLs are presented in Figures 3C,D. From the images, the BHJ blend film on the SnO_2_/PDINO ETL has achieved a more homogeneous surface morphology. The corresponding RMS value is 3.07 nm, which is much lower than that of the BHJ film on the pristine SnO_2_ ETL (4.78 nm). Summing up the findings of AFM, it is suggested that the PDINO modification layer effectively reduced the surface defects of SnO_2_ NPs. Moreover, as the surface morphology of the ETL plays a crucial role in the formation of the BHJ, the surface morphology of PM6:Y6 blend film is consequently improved after the PDINO modification (Peng et al., 2020).

**FIGURE 4:**
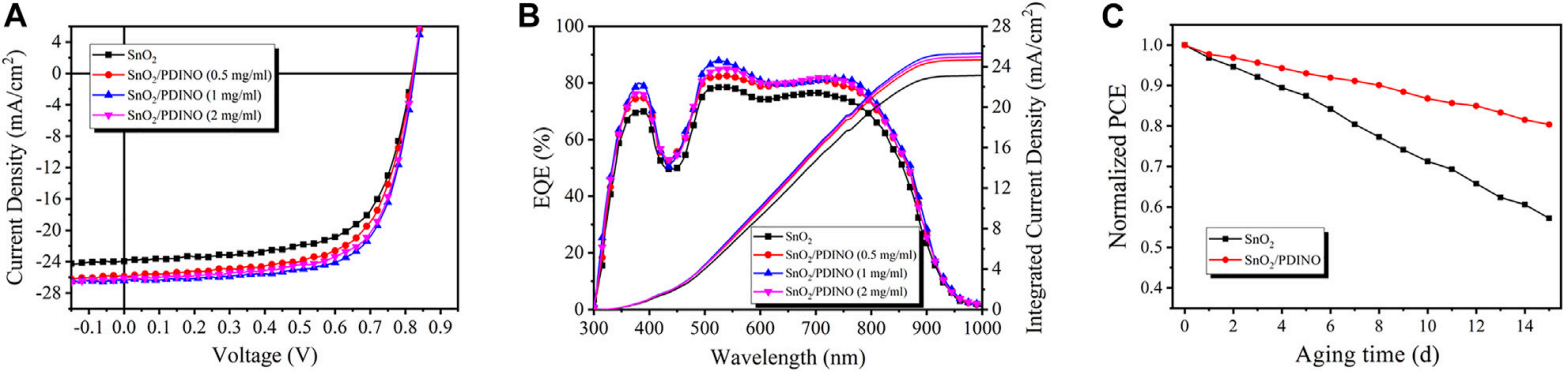
**(A)** *J*–*V* curves of OSCs. **(B)** EQE spectra and JEQE curves of OSCs. **(C)** Normalized PCEs of OSCs after 15 days of aging. The devices were stored in the air at room temperature (∼20°C) and without encapsulation.

To verify the effectiveness of PDINO as a SnO_2_ ETL modifier, the PM6:Y6 OSCs were fabricated based on the SnO_2_ ETL and SnO_2_/PDINO ETLs, with the device structure shown in Figure 1B. The *J-V* curves are recorded and shown in Figure 4A. The performance metrics of the OSCs are summarized in Table 1. The unmodified device with pristine SnO_2_ NPs as the ETL provides a PCE of 12.73%, with an open circuit voltage (*V*_*OC*_) of 0.820 V, a *J*_*SC*_ of 23.95 mA/cm^2^, and an FF of 64.82%. With the introduction of the PDINO modification layer, significant device performance improvements are observed. When PDINO concentration is 1 mg/ml, the champion device reaches its maximum PCE value of 14.97%, with an increased *V*_*OC*_ of 0.825 V, a *J*_*SC*_ of 26.40 mA/cm^2^, and an FF of 68.70%. The enhanced parameters could be ascribable to the better qualities of the SnO_2_/PDINO ETL film and preferable ETL/active layer interfacial contact. When the concentration of PDINO increases to 2 mg/ml, the denser PDINO layer may have an adverse effect on charge carrier transportation and further affect the transmittance of the ETL. Therefore, the device suffers from a moderate performance decrease, with a reduced PCE of 14.58%, a *V*_*OC*_ of 0.825 V, a *J*_*SC*_ of 26.10 mA/cm^2^, and an FF of 67.49%.

**TABLE 1:** Photovoltaic performance metrics of OSCs with pristine SnO_2_ and different concentrations of PDINO-modified SnO_2_ as ETLs. The average PCE (PCEavg) value was obtained form 20 devices fabricated in parallel.

ETL	*V*_oc_ (V)	*J*_sc_ (mA/cm^2^)	FF (%)	PCE_max_ (%)	PCE_avg_ (%)	*J*_EQE_ (mA/cm)
SnO_2_	0.820	23.95	64.82	12.73	12.59 ± 0.06	23.14
SnO_2_/PDINO (0.5 mg/ml)	0.821	25.88	65.43	13.89	13.78 ± 0.11	24.68
SnO_2_/PDINO (1 mg/ml)	0.825	26.40	68.70	14.97	14.73 ± 0.24	25.31
SnO_2_/PDINO (2 mg/ml)	0.823	26.10	67.94	14.58	14.32 ± 0.16	24.97

The measurement of external quantum efficiency (EQE) was also carried out to illustrate the effects of the PDINO modification layer. The EQE spectra are presented in Figure 4B. Compared with the pristine SnO_2_ ETL device, the EQE values of OSCs with SnO_2_/PDINO ETLs notably increased throughout the 350–900 nm wavelength range. For the device with 1 mg/ml PDINO-modified ETL, the maximum EQE value exceeds 85% around the wavelength of 550 nm. The intensified EQE spectra can be ascribed to the combined effects of faster electron transportation and more favorable interfacial contact. From the EQE spectra, the integrated current density (*J*_*EQE*_) curves are plotted in Figure 4B, and the *J*_*EQE*_ values are listed in Table 1. The trend of *J*_*EQE*_ makes a good match within less than 5% of the variation of the corresponding *J*_*SC*_.

As an essential parameter for commercialization in the future, the ambient stability of OSCs was tested in the air at room temperature without encapsulation. From Figure 4C, after 15 days aging, the pristine SnO_2_ NP ETL device exhibited unsatisfying stability, with its PCE dropping to less than 60% of the initial value. In contrast to the control device, the OSC with SnO_2_/PDINO ETL successfully maintained up to 81% of its original PCE. It is suggested that by reducing the possible defects of the SnO_2_ NP layer, the PDINO layer could strengthen the ETL’s oxygen/water shielding effects, thus enhancing the stability of the SnO_2_/PDINO ETL OSC.

**FIGURE 5:**
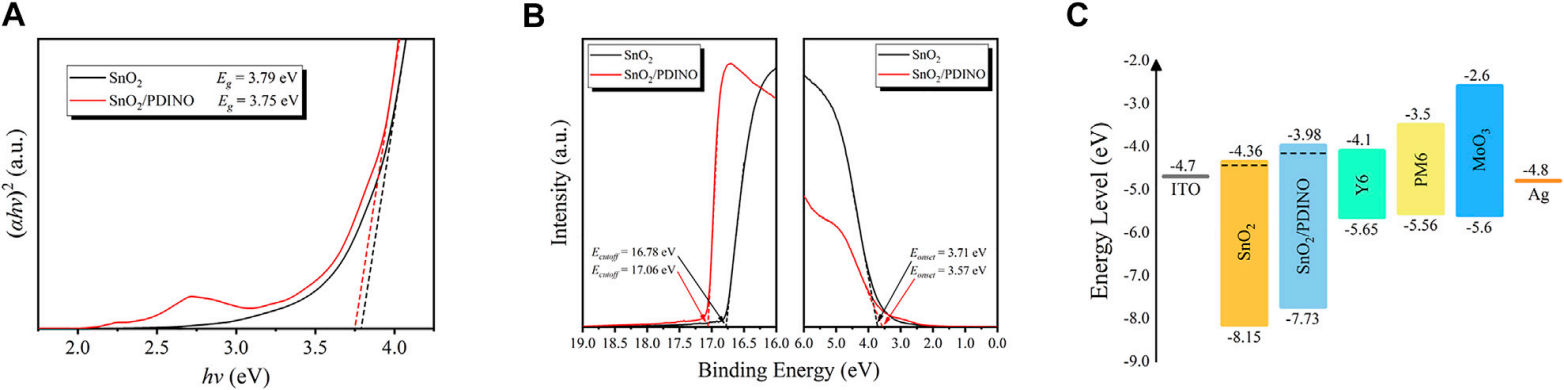
**(A)** Tauc plots of SnO_2_ and SnO_2_/PDINO films. **(B)** UPS spectra of SnO_2_ and SnO_2_/PDINO. **(C)** Schematic diagram of energy levels of OSCs with different ETLs.

To investigate the energy level of the SnO_2_ NP ETL before and after PDINO modification, in Figure 5A, the optical bandgap (*E*_*g*_) was first determined by Tauc plots derived from the UV–vis absorption spectra. The *E*_*g*_ of the pristine SnO_2_ and SnO_2_/PDINO are estimated to be 3.79 and 3.75 eV, respectively. The ultraviolet photoelectron spectroscopy (UPS) measurements were subsequently implemented to determine the binding energies of the cutoff region (*E*_*cutoff*_) and onset region (*E*_*onset*_), which are depicted in Figure 5B. With the incident photon energy value (*hν*) of 21.22 eV (He I), by subtracting the *E*_*cutoff*_ from *hν*, the WF is estimated to be 4.44 eV for pristine SnO_2_ and 4.16 eV for SnO_2_/PDINO. The valence band maximum (VBM) level can be calculated with Eq. 2:

$$\text{VBM}=hv-\left(E_{cutoff}-E_{onset}\right).$$ (2)

For the pristine SnO_2_ and SnO_2_/PDINO, the VBM levels are 8.15 and 7.73 eV, respectively. Considering the *E*_*g*_ of different ETLs, the corresponding conduction band minimum (CBM) levels are 4.36 and 3.98 eV, respectively. From the analyses above, the energy level diagram for the OSC is plotted in Figure 5C. The reduced WF and energy level shifts of the modified ETL can benefit both electron extraction and charge collection abilities, contributing to significantly improved *J*_*SC*_, FF, and the slightly increased *V*_*OC*_ as summarized in Table 1 (Guang et al., 2021).

**FIGURE 6:**
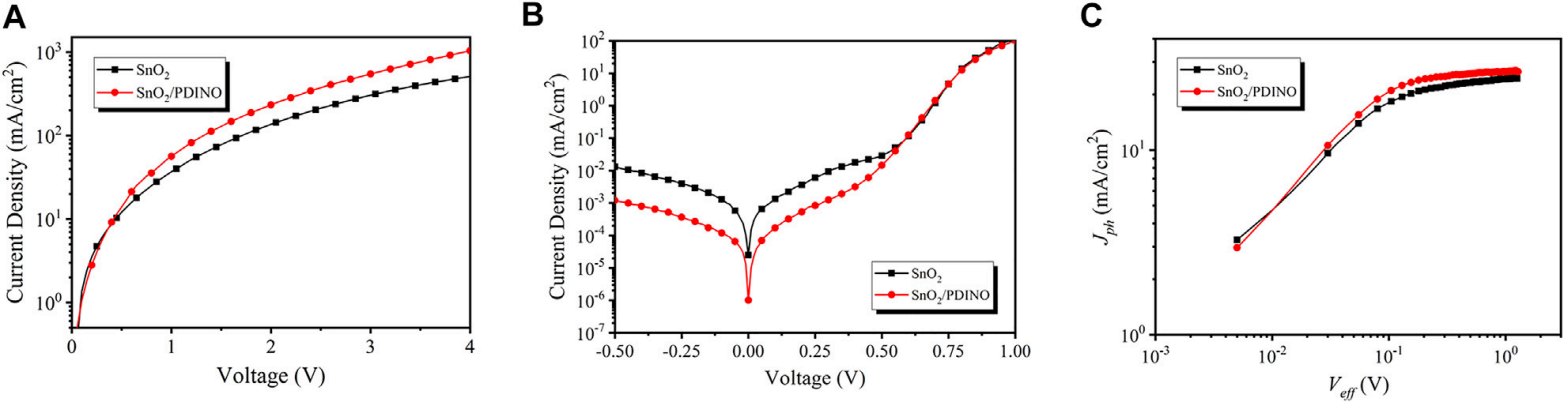
**(A)** *J*–*V* curves of electron-only devices. **(B)** *J*–*V* curves of OSC devices in dark condition. **(C)** Plots of photocurrent (*J*_*ph*_) dependence on effective voltage (*V*_*eff*_).

To conduct an in-depth investigation into the performance enhancements after applying the PDINO layer, the electron-only devices were fabricated and the space charge limited current (SCLC) approach was carried out. The corresponding device structure is ITO/ETLs/PM6:Y6/bathophenanthroline/Ag. The *J-V* characteristics are shown in Figure 6A. Following the Mott–Gurney law, it can be calculated that the electron mobility of devices with the pristine SnO_2_ ETL is 1.14 × 10^−4^ cm^2^/Vs. The electron mobility of the modified device notably increases to 1.92 × 10^−4^ cm^2^/Vs after the introduction of PDINO, indicating that the electron transporting ability of the ETL can be improved effectively. The *J-V* curves in the dark condition of OSCs with SnO_2_ and SnO_2_/PDINO ETLs are displayed in Figure 6B. In the applied voltage range of −0.5–0 V, restrained reverse leakage currents were obtained for the SnO_2_/PDINO ETL device, which suggested that the PDINO modification layer could suppress the charge carrier recombination. From the dark *J-V* characterization, the photocurrent (*J*_*ph*_) dependence on the effective voltage (*V*_*eff*_) is plotted in Figure 6C. *J*_*ph*_ is set as *J*_*ph*_ = *J*_*light*_−*J*_*dark*_, where *J*_*light*_ is the illuminated device current density with the irradiance of 100 mW/cm2 and *J*_*dark*_ is the current density in the no-light condition. *V*_*eff*_ is defined by *V*_*eff*_ = *V*_*0*_−*V*, where *V*_*0*_ refers to the compensative voltage where *J*_*ph*_ (*V*_*0*_) = 0, and *V* is the bias voltage. At a large reverse bias voltage, the saturation current density (*J*_*sat*_) will nearly no longer increase and reach a maximum value. The *J*_*sat*_ for SnO_2_ and SnO_2_/PDINO ETL devices are determined with the values being 24.52 and 26.91 mA/cm^2^, respectively, where *V*_*eff*_ = 1.2 V. The charge collection probability [*P* (E,T)] can be derived from Eq. 3 (Kyaw et al., 2013):

$$P\left(E,T\right)=\frac{J_{ph}}{J_{sat}}.$$ (3)

At the short-circuit (*J*_*ph*_ = *J*_*SC*_) and the maximum power output (*J*_*ph*_ = *J* (*V*_*eff*_ = 0.2 V)) conditions, the *P* (E,T)s were 0.977/0.857 and 0.981/0.899 for the SnO_2_ ETL and SnO_2_/PDINO-based devices, respectively. The larger *P* (E,T) suggests that PDINO modification can promote exciton dissociation and charge extraction of the devices, which is the main responsibility for achieving higher *J*_*SC*_ and FF.

Alongside exciton dissociation and charge extraction, the charge recombination behavior also has considerable impacts on the device performance. Therefore, the *J-V* characterizations under different incident light intensities (*P*_*in*_) were performed. The dependence of *V*_*OC*_ on the *P*_*in*_ in OSCs with and without PDINO-modified ETLs can be deduced from Eq. 4 (Cowan et al., 2010):

$$V_{OC}\propto n\frac{kT}{q}\ln\left(P_{in}\right).$$ (4)

**FIGURE 7:**
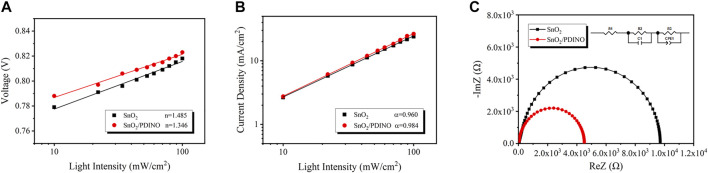
**(A)** *V*_*OC*_ dependence on incident light intensity of the OSCs. **(B)** *J*_*SC*_ dependence on incident light intensity of the OSCs. **(C)** Impedance spectra of the OSCs. An inset of the transmission line model equivalent circuit is in the upper right corner.

The plot of *V*_*OC*_ vs. logarithmic *P*_*in*_ is displayed in Figure 7A. *nkT*/*q* is the fitting line slope. *k* is the Boltzmann constant, *T* is the thermodynamic temperature, and *q* is the elementary charge. When the value of *n* draws closer to 1, it indicates that bimolecular recombination is the dominant recombination process. If the value of *n* approaches 2, the trap-assisted recombination will govern the recombination mechanism. The pristine SnO_2_ ETL device possesses a slope of 1.485 *kT*/*q*, suggesting the existence of trap-assisted recombination. After utilizing the PDINO modification layer, the trap-assisted recombination is restricted as the slope of the modified OSC reduces to 1.346 *kT*/*q*, which distinctly indicates the optimization of surface defects for the SnO_2_ NP ETL (Wu et al., 2019; Yang et al., 2021). To further analyze the bimolecular recombination, the dependence of *J*_*SC*_ on the *P*_*in*_ was evaluated from Eq. 5 (Khlyabich et al., 2012):

$$J_{SC}\propto P_{in}^{\alpha },$$ (5)

where *α* is an exponential factor acquired from the fitting line slope of the *J*_*SC*_–*P*_*in*_ double logarithmic plot. Generally, an *α* value closer to one suggests that there is weaker bimolecular recombination in the OSC. Depicted in Figure 7B, the *α* value of the OSC using the pristine SnO_2_ ETL is 0.960, while the SnO_2_/PDINO ETL device shows a higher *α* value of 0.984. The result indicates that the bimolecular recombination of the OSC is remarkably suppressed by PDINO; the increased device FF is explained as well.

Moreover, impedance spectroscopy measurement was implemented to study the electrical contact properties of the OSCs (Tiwana et al., 2011). The Nyquist plots are displayed in Figure 7C. In the equivalent circuit of the transmission line model, *R*_*1*_, *R*_*2*_, and *R*_*3*_ correspond to the device series resistance, interfacial resistance, and recombination resistance, respectively (Yang et al., 2017). Derived from the Nyquist plots, the resistance metrics are listed in Table 2. The *R*_*1*_ values of the OSCs based on different ETLs are comparable. For SnO_2_/PDINO ETL–based devices, *R*_*2*_ is smaller than half of the value of a pristine ETL-based device, which suggests the improved charge transportation ability in the active layer/ETL interface. Meanwhile, given credit to the optimization of PM6:Y6 morphology, the decreased value of *R*_*3*_ indicates that the charge recombination is effectively suppressed for the active layer deposited upon the SnO_2_/PDINO ETL. Conclusively, these above merits lead to improved *J*_*SC*_ and FF, which eventually enhance the PCE of the OSC.

**TABLE 2:** Resistance metrics of the equivalent circuit for OSCs based on different ETLs.

ETL	R_1_ (Ω)	R_2_ (KΩ)	R_3_ (Ω)
SnO_2_	48.1	9.4	218.5
SnO_2_/PDINO	48.2	4.3	118.7

## Conclusion

In conclusion, a facile SnO_2_ NP ETL modification strategy by utilizing PDINO has been successfully implemented. The introduction of PDINO as a modifier could reduce the surface defects generated by the solution-based fabricating process of the SnO_2_ NP ETL and simultaneously regulate the formation of the active layer’s morphology. For the devices based on the PDINO-modified SnO_2_ NP ETL, the optimized exciton dissociation, enhanced charge collection efficiency, and suppressed molecular recombination synergistically boost the device performance. With enhanced device stability, the corresponding OSCs exhibit a maximum PCE approaching 14.9%, which improved by 17% from the pristine SnO_2_ devices (12.9%). Overall, this work reveals a promising pathway to modify the SnO_2_ NP ETL for achieving high-efficiency and stable OSCs.

## Data Availability

The original contributions presented in the study are included in the article/Supplementary Material; further inquiries can be directed to the corresponding authors.
